# Change in Waist Circumference With Continuous Use of a Smart Belt: An Observational Study

**DOI:** 10.2196/10737

**Published:** 2019-05-02

**Authors:** Myeonggyun Lee, Jaeyong Shin

**Affiliations:** 1 Division of Biostatistics, Department of Population Health and Environmental Medicine New York University School of Medicine New York City, NY United States; 2 Department of Preventive Medicine Ajou University School of Medicine Suwon Republic of Korea; 3 Institute of Health Services Graduate School Yonsei University Seoul Republic of Korea; 4 Department of Policy Analysis and Management College of Human Ecology Cornell University Ithaca, NY United States

**Keywords:** smart health care, wearable device, obesity, internet of things, mHealth, digital health care, lifestyle modification, metabolic syndrome

## Abstract

**Background:**

Health insurers and policymakers are trying to prevent and reduce cardiovascular diseases due to obesity. A smart belt that monitors activity and waist circumference is a new concept for conquering obesity and may be a promising new strategy for health insurers and policymakers.

**Objective:**

This preliminary study evaluated whether the use of a smart belt was associated with a decrease in waist circumference.

**Methods:**

In the manufacturer’s database, there were data on a total of 427 men at baseline. A total of 223, 81, and 27 users kept using the smart belt for 4, 8, and 12 weeks, respectively. Paired *t* tests and repeated measures analysis of variance (ANOVA) were used to identify the change in waist circumference at specified time intervals (at 4, 8, and 12 weeks). In addition, a linear mixed model was used to incorporate all users’ waist circumference data at each time point. Preexisting data on waist circumference and self-reported demographics were obtained from the manufacturer of the smart belt (WELT Corporation, South Korea).

**Results:**

Compared with baseline, the waist circumference (cm) decreased significantly at all time points: –0.270 for week 4, –0.761 for week 8, and –1.972 for week 12 (all *P*<.01). Although each paired *t* test had a different sample size because of loss to follow-up, the differences between baseline and each subsequent week increased. Equal continuous reduction in waist circumference was observed with the ANOVA and mixed model analysis (beta=–0.158 every week).

**Conclusions:**

The smart belt is a newly developed, wearable device that measures real-time steps, sedentary time, and waist circumference. In this study, we showed that wearing the smart belt was associated with reducing waist circumference over 12 weeks. This direct-to-consumer smart health device may contribute toward reducing the risk of obesity and related conditions and controlling increasing health costs for health insurers.

## Introduction

Noncommunicable diseases (NCDs) are one of the leading causes of death worldwide [1-3]. According to the World Health Organization, of the 56.4 million global deaths in 2015, 39.5 million (70%) were due to NCDs [4]. Currently, the burden of NCDs is growing faster than our ability to combat them because of the obesity epidemic [5-7]. In the United States, the percentage of the national medical expenditures devoted to treating obesity-related illness in adults increased from 6.13% in 2001 to 7.91% in 2015 [8].

Due to a change to a Western lifestyle, including a high-calorie diet, and the aging society in Korea, the epidemiology of NCDs in Korea has dramatically risen. According to national statistics, the age-adjusted prevalence rate of metabolic syndrome in Korea has increased from 21.6% in 2007 to 26.5% in 2015 among men, whereas there has been slight decrease among women from 20.3% to 17.6% during the same period [9]. Based on forecasting models including these variables, the 2020 and 2030 estimates for obesity prevalence in Korea are 47% and 62% for men and 32% and 37% for women, respectively [10]. Therefore, it is necessary to control unhealthy lifestyles in Korea.

Several insurers and health policymakers have attempted to prevent NCDs and their related lifestyles [11-13]. However, changing lifestyles to prevent NCDs is not easy. Thus, new concepts to conquer NCDs have been introduced and are being tested. For example, the Centers for Disease Control and Prevention (CDC) approved some digital health programs as part of the National Diabetes Prevention Program. Diabetes can be controlled and prevented by lifestyle modification. Therefore, a key part of the National Diabetes Prevention Program is the lifestyle change program, which aims to prevent or delay type 2 diabetes [14,15]. The CDC recognizes lifestyle change programs that meet certain standards and show that they can achieve results. These standards include following an approved curriculum, being facilitated by a trained lifestyle coach, and submitting data each year to show that the program has had an impact. However, only a few wearable devices included in the programs satisfy these standards. In addition, most of them measure daily activities and self-reported diet and body mass index (BMI) [16].

**Figure 1:**
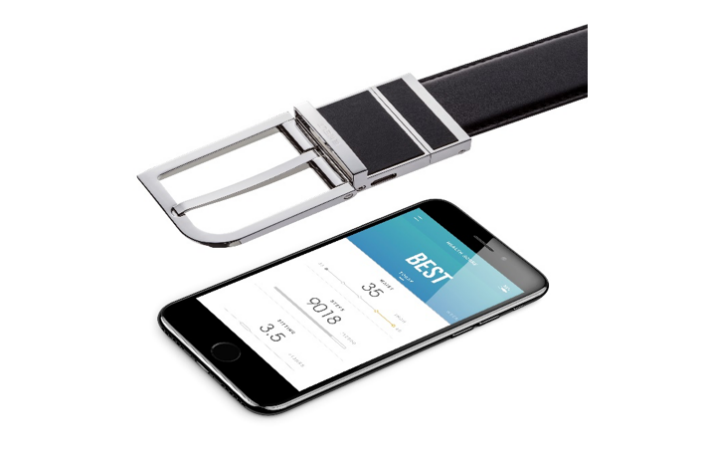
The WELT smart belt and mobile phone app.

The WELT smart belt was launched in 2016 by the WELT Corporation, which is a health care technology company. The smart belt looks like a normal belt but can monitor a wide range of health data using a mobile phone app. It can measure an individual’s waist circumference, overeating habits, number of steps, and sedentary time with the tracking technology stored in the belt buckle (see the image in Figure 1) to help improve the individual’s health and the effectiveness and the efficiency of the health care system [17]. From the collected information, the device provides users with their daily activity score categorized into three groups: best, good, and poor. All real-time information is presented on a mobile phone app, and comparisons between daily activities and the previous days’ activities are possible. However, the device includes no feedback or notification that encourages exercise or warns of overeating.

This preliminary study aims to evaluate whether the waist circumference of smart belt users decreased with use. Moreover, this device has a distinct measurement approach, which other smart devices do not have. The waist circumferences can be measured every 30 minutes, 24 hours per day, and these measurements are saved into a database as a longitudinal concept. Thus, to the best of our knowledge about the smart belt, and because none of the previous studies have shown this type of user waist data from the smart wearable device, evaluating its effectiveness is necessary.

## Methods

### Study Population and Variables

**Figure 2:**
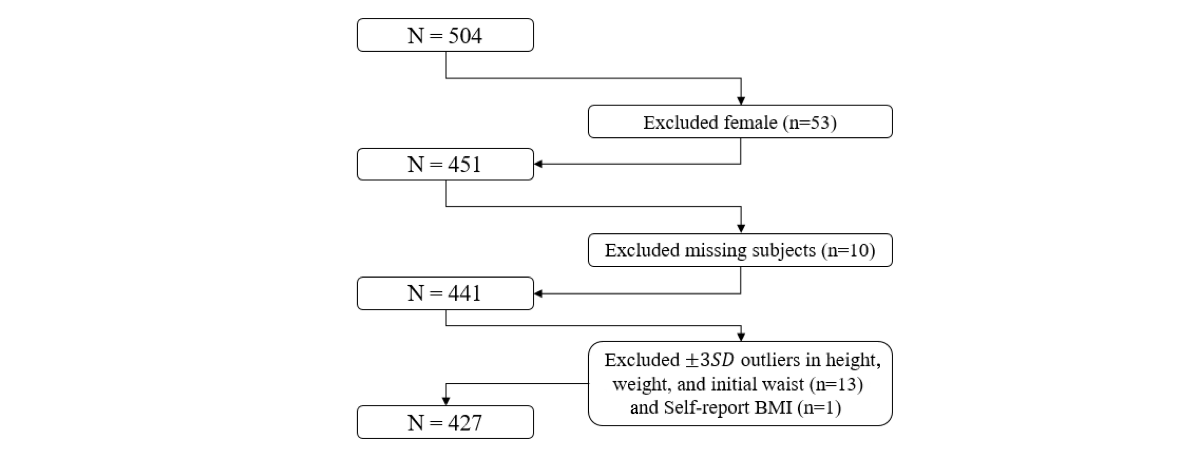
Flowchart for study participants. Preexisting data were obtained from the WELT Corporation. BMI: body mass index.

This study included smart belt users who downloaded the app on their mobile phones and provided information on their weight, height, and age. When they downloaded the app, the WELT cooptation asked them to monitor their health variables without identification. The app measures the waist circumference of the user wearing the smart belt at 30-minute intervals and provides information on waist circumference, steps, and sedentary time. Preexisting data were obtained from the WELT Corporation from July 2016. A total of 451 male users were registered in the database initially, and 24 who had missing values or were outliers for height, weight, and initial waist circumference were excluded (Figure 2). This study was approved by the institutional review board at Yonsei University in Korea (approval number: Y2018-0264-001).

In the database, 504 subjects were registered initially, and we excluded 77 who were female (n=53), missing data (n=10), or outliers in height, weight, and initial waist circumference (n=14). Finally, we obtained a total number of 427 users’ data.

**Figure 3:**
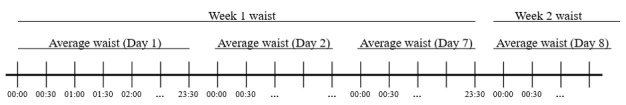
Calculations of daily and weekly waist circumferences. The first week was the baseline measure.

The daily and weekly waist circumferences (cm) of the users were calculated using an average value of the waist circumference data measured every 30 minutes (Figure 3). If a person did not wear the smart belt, the circumference was recorded as a missing value in our dataset. Therefore, we knew when the users started to wear the smart belt and when they took it off. The waist circumference measurements were obtained when it was worn. We calculated the average waist circumferences without missing values. The average waist circumference after the first week of wearing the smart belt and using the app was considered the baseline measurement. This study reports the waist circumference of users during the first 12 weeks of using the smart belt.

### Statistical Analysis

From the 427 user datasets, descriptive statistics were calculated for the users’ baseline characteristics, and follow-up waist circumference measurements were reported as means with standard deviations. There were losses to follow-up on wearing time; thus, the sample size decreased at 12 weeks compared with baseline.

A paired *t* test is generally performed to compare different conditions among the same subjects; this approach has been used elsewhere in public health research [18]. In this study, a paired *t* test was performed to identify the change in waist circumference between baseline and each follow-up week for the same subjects.

As a multivariate approach, a repeated measures analysis of variance (ANOVA), used to distinguish the influence of time on waist circumference change [19], was used to identify the change in waist circumference by considering time intervals. We used the following time intervals: every week, biweekly, and at 4-week intervals. Although this method extracts the contribution of subjects from the error term, some subjects were excluded from the entire analysis if the values for even one time point were missing.

A linear mixed model analysis, which incorporated all subjects’ waist circumference data at each time point, was conducted. A linear mixed model was used to identify the trend of the findings of previous public health studies by Xu [20], De Onis et al [21], and Brodersen et al [22]. The following models were considered: using only week time (model 1), using week time and BMI (model 2), and using adjusted time and baseline variables (model 3). Model 3 consisted of covariates from model 2 as well as age and wearing time (days) as an adjustment and attachment, respectively.

Based on the duration of continuous smart belt use, the users were divided into three groups: initial only (used the belt in the first week), passive users (used the belt between 2 and 5 weeks), and active users (used the belt for more than 5 weeks). ANOVA was used to compare the demographic characteristics and initial waist circumference of the three groups. Significance tests were two-sided, with the significance level set at .05. Multiple comparison problems were not considered because this was an observational study and our purpose was to identify the trend in waist circumference change in subjects who wore the smart belt. All statistical analyses were performed using SAS 9.4 software (SAS Institute Inc, Cary, NC, USA).

## Results

Table 1 shows the descriptive characteristics of the smart belt users. To calculate statistics, we divided the followed-up users as (1) all cases, (2) completed users up to the first 4 weeks, (3) completed users up to the first 8 weeks, and (4) completed users up to 12 weeks. For example, if one user dropped out at week 6, data from that user was used for “up to 4 weeks” but not “up to 8 weeks” or later. From the demographics, the population was representative of Korean, middle-aged, male, obese office workers based on age, weight, height, and BMI.

**Figure 4:**
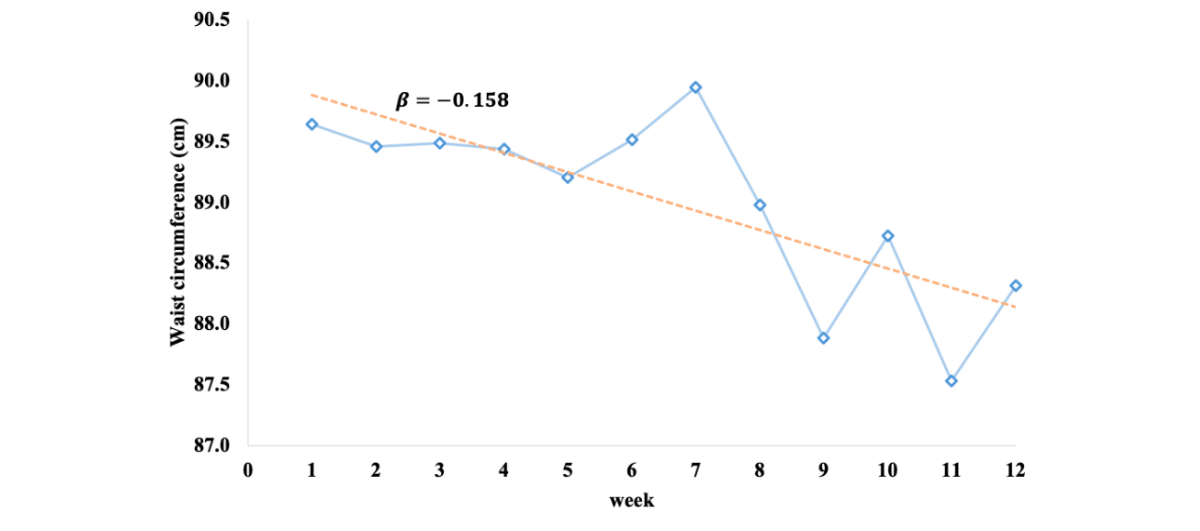
Trend of waist circumference by week. The blue solid line is the mean value of the waist size for each week; the orange line is a linear trend of the waist size by week.

**Figure 5:**
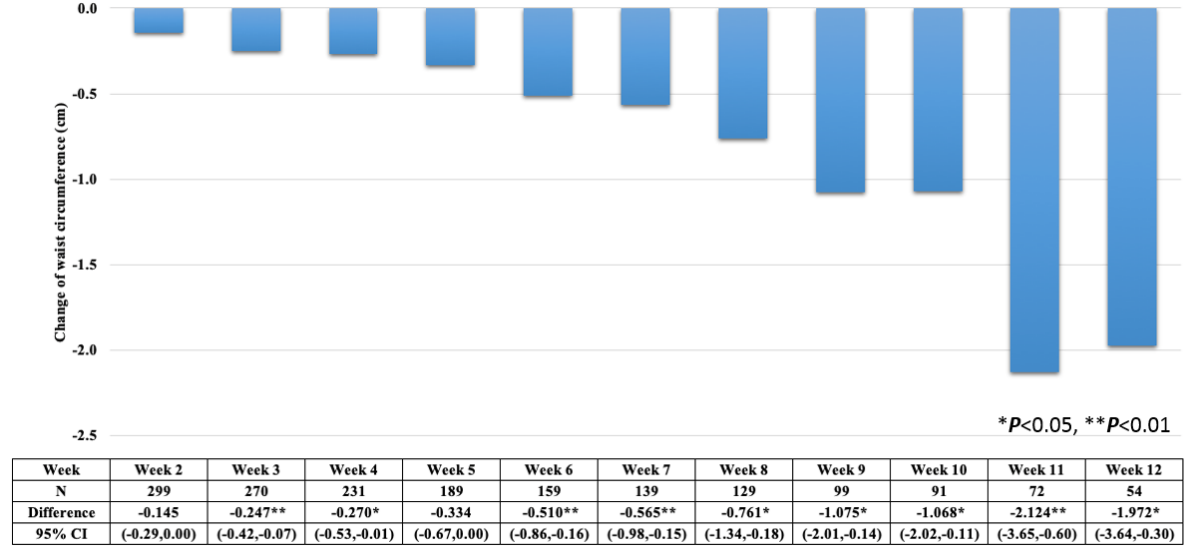
Reduction in waist circumferences of users each week over 12 weeks compared to baseline using paired *t* test.

Average waist circumference decreased a mean 0.158 (SD 0.043) cm every week from baseline to the last followed-up week (Figure 4). To compare each week with the baseline, a paired *t* test was conducted. Since users who abnormally increased or decreased their waist size could be outliers, we excluded those outliers with a cutoff of 3 standard deviations from the mean for each comparison. Mean waist circumference decreased at all time points (all weeks) compared to the baseline measurement, with *P* values for the difference between baseline and each time point of *P*<.05 except for weeks 2 and 5 (Figure 5). Although each paired *t* test had a different sample size because of the loss to follow-up, the magnitude of difference between the baseline value and the value at each subsequent week until the final follow-up week increased (Figure 5). With the decreasing trend of waist size in Figure 5, the increment of waist size at week 7 in Figure 4 could be caused by outliers with cutoffs 3 standard deviations from the mean.

**Table 1:** Descriptive statistics of smart belt users. Except for waist circumference, other characteristics, including age, weight, height, and body mass index (BMI), were self-reported at baseline when the user first started using the app.

Characteristic		All (N=427), mean (SD)	Used up to 4 weeks, (n=223), mean (SD)	Used up to 8 weeks, (n=81), mean (SD)	Used up to 12 weeks, (n=27), mean (SD)
Age (years)		42.29 (11.83)	44.45 (12.35)	45.17 (12.62)	43.11 (12.97)
Weight (kg)		79.80 (12.86)	79.65 (12.72)	78.65 (13.06)	75.07 (12.19)
Height (m)		1.75 (0.07)	1.74 (0.07)	1.75 (0.08)	1.72 (0.07)
BMI (kg/m^2^)		25.89 (3.47)	25.96 (3.38)	25.54 (3.48)	25.25 (3.40)
**Waist (cm)**					
	Week 1 (n=427)	89.64 (9.04)	89.38 (8.79)	89.41 (8.71)	89.79 (7.29)
	Week 2 (n=303)	89.46 (9.19)	89.48 (8.94)	89.46 (8.66)	89.66 (7.95)
	Week 3 (n=276)	89.48 (9.04)	89.28 (8.99)	89.31 (8.64)	89.43 (7.87)
	Week 4 (n=239)	89.43 (9.04)	89.23 (9.04)	89.18 (8.76)	89.48 (7.77)
	Week 5 (n=195)	89.20 (9.07)		88.85 (9.09)	89.23 (7.82)
	Week 6 (n=165)	89.51 (8.71)		89.20 (8.71)	89.03 (7.95)
	Week 7 (n=146)	89.94 (8.33)		88.65 (8.92)	87.86 (8.76)
	Week 8 (n=134)	88.98 (9.22)		88.37 (9.19)	87.55 (9.14)
	Week 9 (n=103)	87.88 (8.97)			87.60 (8.97)
	Week 10 (n=94)	88.72 (8.81)			87.48 (9.09)
	Week 11 (n=74)	87.53 (9.83)			87.10 (9.63)
	Week 12 (n=55)	88.32 (9.27)			86.89 (9.68)

Change in waist circumference was analyzed using repeated measures ANOVA. We considered three models at different time intervals (Table 2). Model 1 shows that the differences in waist circumference at weeks 2, 3, and 4 were mean –0.157 (SD 1.260), mean –0.254 (SD 1.364), and mean –0.325 (SD 1.816) cm, respectively. The differences decreased by week. In the case of model 3 as a four-week interval, decreasing waist circumference was more distinct; there was an average decrease of 1.435 (SD 4.442) cm between baseline and week 12. Since the objective was to compare each week as a group, we included all users in which they wore the smart belt each week and the data were recorded, even though they may not have followed up at all time points. Although Table 1 shows the completed users (eg, the 27 users for “used up to 12 weeks” means that they completely followed up), we used users who were followed up at weeks 1, 4, 8, and 12 in the repeated measures ANOVA. Thus, the number of users could be different. Although each model had a different sample size because of loss to follow-up, all three models showed a *P* value less than .05 under the null hypothesis that the differences of the waist circumference would not differ for each week compared to baseline. Thus, all three models had a statistically significant change in waist circumference, with the mean waist circumference decreasing by week.

Repeated measures ANOVA could be used to determine the change in waist circumference; however, in this study, only the data of users with complete information at the measuring points (ie, 4, 8, and 12 weeks) were included. Thus, an additional analysis that incorporated all the observed waist circumferences was necessary to identify overall waist circumference reduction. Table 3 shows the results of the linear mixed model using all datasets. The results show a decrease in waist circumference regardless of adjustments.

**Table 2:** Differences in waist circumference using repeated ANOVA for three models.^a^

Model		Difference from baseline, mean(SD)	*P* value	Cohen *f*
**Model 1: weeks 1, 2, 3, 4 (n=215)**			.002	0.1532
	Week 2	–0.157 (1.260)		
	Week 3	–0.254 (1.364)		
	Week 4	–0.325 (1.816)		
**Model 2: weeks 1, 2, 4, 8 (n=111)**			.003	0.2065
	Week 2	–0.157 (1.260)		
	Week 4	–0.254 (1.364)		
	Week 8	–0.325 (1.816)		
**Model 3: weeks 1, 4, 8, 12 (n=41)**			.03	0.2744
	Week 4	–0.173 (1.402)		
	Week 8	–0.762 (3.584)		
	Week 12	–1.435 (4.442)		

^a^ Note that each model has a different sample size due to loss at follow-up.

**Table 3:** Adjusted models for waist circumference using the linear mixed model.^a^

Variable	Model 1			Model 2			Model 3		
	Estimate	Standard error	*P* value	Estimate	Standard error	*P* value	Estimate	Standard error	*P* value
Intercept	89.939	0.442	<.001	64.302	3.018	<.001	63.455	3.381	<.001
Week	–0.142	0.026	<.001	–0.141	0.026	<.001	–0.143	0.026	<.001
BMI^b^				0.990	0.116	<.001	0.999	0.116	<.001
Age							0.006	0.034	.87
Wearing period (days)							0.008	0.010	.39

^a^Change in waist circumference was analyzed through repeated ANOVA using three models with different time intervals. Note that each model had a different sample size because they had a loss to follow-up.

^b^BMI: body mass index.

We also investigated users who used the smart belt for as long as possible. Here, ANOVA was used to compare the following three groups to investigate the difference in characteristics: initial only, passive users, and active users (Multimedia Appendix 1). There were no statistically significant differences in BMI and waist circumference at baseline, but the group of active users was older than the other groups (age variable was the only significant variable).

## Discussion

In our study, waist circumference tended to decrease among smart belt users. In the paired *t* test analysis, the users who wore it longer had greater reductions in waist circumference. The same trend was also found using the linear mixed models. A reduction in waist circumference was observed after each week of device use.

The recent Patient Protection and Affordable Care Act has incentivized physical activity counseling by primary care physicians, which may increasingly affect not only physicians but also health insurers and accountable care organizations. The insurers want to monitor the health behaviors of the insured and to predict the risky health outcomes. Therefore, it is necessary to evaluate the efficiency of newly developed wearable smart health devices and determine how these devices can improve health outcomes.

Various insurers and health-related companies are tapping into wearable technology for their customers to help them stay in shape and to offer their customers wellness discounts and other benefits if they meet a required number of fitness goals [17,23-25]. For example, health insurance start-up provider Oscar provides their customers with a Misfit fitness band to track their physical movements and day-to-day activities—it even monitors the users’ sleep. Based on these data, customers are rewarded once they meet their fitness goals [24]. UnitedHealthcare and Fitbit reward their users with up to US $1500 for activities completed using their device [23]. The companies can track their enrollees’ physical activity and monitor their diet; therefore, they can provide appropriate feedback through personalized wellness programs. Optimal Health’s program differs slightly regarding the incentives because the total reward is paid out incrementally as the participants complete each of the four steps in the challenge, which includes downloading the app and completing a personal health assessment questionnaire, tracking nutrition, wearing the devices, and doing physical activity [23]. Another major health insurance provider, Humana, also provides a 10% annual premium discount to their members if they use fitness tracking devices [23].

Cadmus-Bertram et al [26] performed randomized trials of a Fitbit-based physical activity intervention with 51 inactive postmenopausal women with a BMI greater than 25.0 kg/m^2^ for 16 weeks. Fitbit devices and app-based weight control services were provided to 21 participants in the treatment group (mobile phone app plus Fitbit), whereas the control group used simple pedometers (Fitbit). The participants were encouraged to perform physical activity. The treatment group showed an improvement in moderate-to-vigorous physical activity at 16 weeks compared to those at baseline. However, no statistically significant difference in physical activity between the treatment and control groups was observed.

Jakicic et al [27] compared a standard behavioral weight loss intervention (n=233) and a technology-enhanced weight loss intervention (n=237). The technology-enhanced weight loss intervention group was equipped with commercially available wearable devices, which included Web interface technology. No significant difference between the two groups at 24 months was observed. In these well-designed randomized controlled trials, no reduction in body weight between the group using smart devices and the control group was observed.

According to a systematic review of 22 studies consisting of 16,476 and 14,475 participants in the intervention and control groups, respectively [28], Web-based physical activity interventions had a significantly positive effect on increasing physical activity and daily walking steps among the general population at the initial stage. However, the effect appeared to depend on the design of the study, age of the participants, and duration of the study.

Although controversies regarding the effectiveness and efficiency of smart health devices exist, we believe that our results provide scientific evidence for the effectiveness of the smart belt. Most of the users in our study were obese (BMI >25 kg/m^2^), middle-aged Korean males. Korea has the second-longest working hours according to the Organization for Economic Co-operation and Development (OECD); on average, Koreans work 393 more hours per year than their counterparts in the OECD [29]. Their length of working hours is approximately 1.6 times that of Dutch workers. Thus, exercising regularly is not easy for middle-aged Korean workers. As they may need to check their daily activities and increase them, they would most likely purchase a smart belt for themselves or receive it as a gift from someone, which in turn provides motivation for the smart belt users to lose weight. We could assume that this motivation may be greater in more obese users. Moreover, on the basis of our baseline results, active users who continued to use the smart belt for more than 5 weeks were older than other users. Older people might have more medical conditions and therefore be more interested in their health status, which may explain the high retention in this age group.

However, we still had a fundamental question about the reduction in waist circumference without specific intervention in this study. We speculated that wearing a smart belt device would have a positive effect on one’s health behaviors. Choi et al [30] investigated a 12-week mobile health (mHealth) physical activity intervention for feasibility and potential efficacy. Thirty pregnant women were randomized to either the intervention (mobile phone app plus digital intervention program) or control (digital intervention program alone) group. The difference in weekly mean steps per day was not statistically significant between the groups, which could be because the active control condition (included the use of a wearable activity monitor and final goal steps that were similar to those in the intervention group) may itself have had substantial effects. Even though there is no study on the smart belt, we assume that wearing smart devices including the smart belt possibly motivates users to reduce their waist circumference.

The number of steps taken is a good indicator of an individuals’ sedentary level; this information will provide more details on the characteristics of people who use a smart belt. Although we did not include the results for step count in this study, the mean steps per hour while wearing the device increased over time (see Multimedia Appendix 2). For example, the differences in the mean steps per hour from baseline to weeks 2, 4, and 8 were 220.6, 541.7, and 862.9, respectively. Therefore, it seems that participants in this study had increased activity levels during the 12-week period of observation.

This study has several limitations. First, there was no control group in this study. Therefore, the study participants were motivated individuals who were interested in trying a novel product to help with a lifestyle change such as weight loss. Hence, future studies need to include participants who do not wear the smart belt as a comparison control group. Second, some important variables may have been omitted (for example, regular exercise hours or health conditions that may affect daily activity). In this study, we used only the data collected from the wearable device and self-reported data; that is, this cohort could be more self-driven and determined to change their behavior or lifestyle to lose weight compared to the general population. Thus, this cohort might also seek other weight loss interventions, such as an exercise program, which could contribute as confounding factors during the period of using the smart belt. However, those confounding factors also have some limitations in this study; for example, it is uncommon for a waist belt to be worn with sports attire or active wear when users exercise. Since it might be unclear whether participants wore the smart belt all day long, including when they exercised, further research collecting more accurate data on physical activity levels from users is necessary. Third, the retention rate was low. In our study, the follow-up status of the users depended on their usage of the smart belt. Therefore, our results may be applied mainly to active smart belt users. Moreover, it is limited that there are different characteristics of how long the smart belt was used continuously among participants. Fourth, we have to be careful about reverse causation. The relative long-term smart belt users may have higher unmeasured motivation to reduce waist circumference. However, we were unable to measure the amount of motivation in this study. Thus, a comprehensive measurement of the motivation to decrease waist circumference would be a potential strength in future studies.

Our study also has strengths compared with previous research. Although this study is insufficient to fully validate the effect of the smart belt due to flaws in study design and unmeasured confounding factors, to our knowledge no other studies have reported on the smart belt and its effectiveness. Through this observational study, we examined the association between a new smart wearable belt device and a reduction in waist circumference. Lastly, the smart belt could be used daily by male workers in Korea. Since a dress code is important in the strict and conservative organizational culture in Korea [31], most male employees in Korea usually wear the belt during the weekdays. Therefore, we expect that the smart belt would not involve having to remember to wear an additional device because almost everyone in this demographic has to wear a belt anyway.

A smart belt is a newly developed wearable device that measures real-time steps, sedentary time, and waist circumference. In our study, we showed the smart belt may have an association with a reduction in waist circumference among users through the various quantitative approaches in an observational study. Based on this result, further studies including other confounding factors (eg, change in lifestyle habits and health programs) and a control group to make comparisons should be considered. Therefore, the benefit of this research is that it may be used as the foundation for future related studies. This direct-to-consumer smart health device may contribute toward reducing the risk for NCDs and controlling the increasing health costs for health insurers, but a randomized controlled trial is necessary to further measure its effectiveness.

## Appendix

Multimedia Appendix 1 Comparing the baseline characteristics among the three groups.

Multimedia Appendix 2 Difference between average steps per hour compared to baseline week.

## Abbreviations

NCD: noncommunicable disease
ANOVA: analysis of variance
CDC: Centers for Disease Control and Prevention
BMI: body mass index
OECD: Organization for Economic Co-operation and Development
