# Supplementary material for: Change in Waist Circumference With Continuous Use of a Smart Belt: An Observational Study
Source: JMIR Mhealth Uhealth. 2019 May 2;7(5):e10737. doi: 10.2196/10737 (PMC6521184; doi:10.2196/10737)
Supplement: Multimedia Appendix 1 [file mhealth_v7i5e10737_app1.pdf]

Supplementary Table 1. Comparing the baseline characteristics among three groups (Mean±SD)

Variable	Initial only (N=101)	Passive user (N=126)	Active user (N=200)	p-value
**Age**	38.68±10.73	41.36±10.55	44.72±12.60	<.0001
**BMI**	25.89±3.83	25.98±3.61	25.83±3.21	0.9338
**Follow up (Day)**	11.22±38.54	20.94±8.23	74.90±34.10	<.0001
**Waist circumference (inches, at baseline)**	35.18±3.96	35.29±3.54	35.35±3.37	0.9332
