# Supplementary material for: Change in Waist Circumference With Continuous Use of a Smart Belt: An Observational Study
Source: JMIR Mhealth Uhealth. 2019 May 2;7(5):e10737. doi: 10.2196/10737 (PMC6521184; doi:10.2196/10737)
Supplement: Multimedia Appendix 2 [file mhealth_v7i5e10737_app2.pdf]

Supplementary Table 2. The difference of the average steps per hour compared to baseline week

Weeks	Number of steps an hour (Mean ± SD)
**Baseline (Week 1)**	1075.3±917.6
**Week 2**	1295.9±1777.7
**Week 3**	1326.0±2623.4
**Week 4**	1624.7±3114.1
**Week 6**	1634.9±2621.5
**Week 8**	1814.4±2876.3
